# Progress in Surgery

**Published:** 1895-09-28

**Authors:** 


### Progress in Surgery.

### BRAIN SURGERY.

(*Continued from page 432.*)

**Surgical Treatment of Epilepsy.**—Eulenburg[9] says that no guiding rules can yet be laid down as to what cases are likely to be benefited by operation, either in traumatic or idiopathic epilepsy, partly on account of the difficulty of estimating the true value of a history of injury, and partly from the impossibility of determining the exact nature of the disease. He quotes various illustrative cases.

**Mastoid Disease.**—Acute mastoiditis is the subject of a clinical lecture by J. Orme Green,[10] apropos of a patient, aged 25, presenting the common history of coryza, pain in one ear and then in the other, accompanied by serous effusion, with rupture of the drum-membranes, and serous discharge from the meatuses, the right becoming purulent. The spontaneous closing of the perforation in the right membrane was followed by greatly increased pain in the ear, and later by tenderness over the mastoid antrum and tip. This was somewhat relieved by paracentesis of the tympanic cavity, but increased again, and an opening was advised in the belief that pus had formed sub-periosteally. At the operation it was found that, although there was great œdema of the tissues and osteitis, there was no actual pus under the periosteum. The antrum contained much septic pus. The writer's experience shows that when relief is given by operation before the formation of pus sub-periosteally the osteitis usually subsided without caries or necrosis. He points out the value of estimating the seat where the osteitis of the mastoid begins (as indicated by the point of tenderness on pressure), with a view to determining the direction of its spread and the risk of involvement of dangerous structures. He considers the temperature chart of little value in the diagnosis of mastoiditis—the absence of a high temperature being no argument against the existence of abundant suppuration.

In a review of the recent work on the surgery of the mastoid, Milligan[11] gives a brief sketch of the history of the operation from Riolanus, who in 1649 first performed it, to Von Troltsch, who placed it on a sound scientific basis, and Schwartze, whose 100 cases, with 74 cures, proved its value. From the surgical as well as the anatomical point of view, Milligan prefers to look upon the mastoid antrum as a backward continuation of the middle ear, rather than as a much-enlarged pneumatic cell, as thus can be explained the frequency of suppurative affections of the ear, spreading to the antrum. Politzer's indications for opening the antrum in cases of chronic mastoiditis are quoted at length, and after discussing the relative merits of the gouge and mallet (as advocated by Von Troltsch) and the dental burr of Macewen, he describes the operations performed by Von Bergmann, Kuster, Stacke, and Schwartze.

For chronic mastoiditis many surgeons seem to prefer the Stacke method to all others. In this operation a free incision is carried close to the back of the auricle, which is then thrown forward, and with a gouge the superior portion of the meatus is cut away freely enough to throw the attic, tympanum, antrum, and meatus into one large cavity, which affords perfect drainage. Scott Bishop[12] deals with the same subject. He prefers Schwartze's operation for primary mastoiditis, and Stacke's for chronic disease with involvement of the antrum.

Dr. Garnault[13] has made a series of craniometric observations to test the truth of Korner's statement that there is greater danger in brachycephalic than in dolicocephalic subjects, of opening the middle fossa in operations involving the petrous temporal bone. He cannot confirm this. There are individual variations irrespective of the configuration of the skull, and he thinks that before intervention all cases must be regarded as dangerous and treated as such.

Broca[14] gives the results of operations on 82 patients, of trephining the apophysis of the mastoid, chiefly in children (80). Ten died within a few days of the operation from intercurrent diseases. The others recovered. In a large proportion where Wilde's incision was used fistulæ remained.

Baker[15] records a unique case of combined suppuration in the antrum of Highmore, and mastoid disease, the latter condition giving rise to the former.

**Cerebral Abscess.**—Several new cases of cerebral abscess successfully treated have recently been recorded. Unsuccessful cases are less in evidence.

From among the examples of traumatic abscess may be quoted the following: A young American coal miner was struck on the back of the head by a falling slate, and his forehead was forced forward against an iron bolt, which caused a severe scalp wound and compound fracture of the frontal bone above the left orbit. Unfortunately, the scalp wound was stitched up and the patient was allowed to go out. When McComas[16] first saw him 69 days after the accident the scalp wound was still open and a fracture could be readily made out. He complained of pain in his head, and his cerebration was slow. Pulse was 58, temperature 101·4, and respiration 23. Two days later he had a convulsion and became comatose. An operation was performed, and a piece of bone found driven into the brain was removed, and about three ounces of pus escaped from an abscess in the frontal lobe. During the after treatment a hernia cerebri formed, and was removed by transfixing its base with a needle armed with a double thread, tying it in two halves, and shaving off the mass. When seen fifteen months later he was in good health, and to all appearance mentally sound.

Nasse[17] records two cases: (1) A man, 43, eleven days after receiving a perforating wound of the skull, developed slight aphasia and twitching of the mouth, followed by a slight rise of temperature and a slow pulse, with apathy. An abscess was found in front of the anterior central convolution and evacuated. The patient slowly recovered. (2) A boy, six, had a nail driven into his head by a falling log. He developed symptoms closely corresponding to the last case, and on an operation being performed, an area of softening, with splintered bone, was found and removed. Cessation of symptoms and recovery followed.

A fatal case of cerebellar abscess, reported by Purvis[18] is worthy of note. The points of special interest in the case were: (1) there was meningitis co-existing; (2) while the otorrhœa had been present for years, the abscess in the cerebellum was acute both in symptoms and morbid anatomy; (3) optic neuritis was definitely absent; (4) headache was very severe; (5) there were no rigors; (6) temperature, pulse, and respiration were all higher than usual; (7) there was great mental irritability; (8) rigidity of the neck muscles was marked, but the head was not retracted. Post-mortem it was found that the cerebellum was adherent to the posterior surface of the petrous portion of the temporal on the right side, and on freeing it an abscess burst. This was two inches in diameter and contained very offensive pus. The idea of operating had been abandoned on account of the weak condition of the patient.

**Sinus Thrombosis.**—Battle[19] communicates a paper on this subject, based on three cases treated by himself, which illustrate very typically the varying clinical features of this serious complication of middle ear disease. The points on which Macewen relies in diagnosing this condition are quoted by Battle. "In a case of chronic otitis media the cessation of otorrhœa coincident with accession of persistent otalgia, extending into cephalalgia, high temperature with marked fluctuations, vomiting, and rigors, ought to be regarded as pointing in the direction of thrombosis of the lateral sinus." In one of his cases there was no history of rigors or vomiting, the ear discharge continued, and the point of greatest tenderness was above the mastoid. In this patient, however, a cerebellar abscess coexisted with the sinus thrombosis. The writer lays stress on the value of the tender swelling along the line of the internal jugular vein, accompanied by feverishness, with or without rigors, as an important diagnostic point, especially when there is no glandular enlargement. On the other hand, he considers that excessive tenderness on pressure over the position of the mastoid foramen is of less diagnostic value than some are inclined to attach to it. In two of his cases it was absent. He also calls attention to the vertebral veins as possible courses of septic emboli to the lungs.

The indications in operating are (1) to remove the bony wall of the sigmoid sinus and evacuate any pus, whether outside the lateral sinus or pent up with septic clot in the interior of the venous sinus; (2) to block the main channel along which the emboli would travel by means of ligature after section of the internal jugular vein; (3) to remove clot from sinus or vein as far as possible; (4) to clear the mastoid cells; (5) to treat complications as they arise; (6) to operate as soon as possible.

Milligan[20] also writes on this subject. After drawing attention to the anatomical arrangement of parts in explanation of the spread of the septic process from the middle ear to the mastoid process and thence to the venous sinuses, especially the sigmoid sinus, he mentions that thrombosis of that sinus is commonest between the ages of twenty and thirty, while it is rare in children and old people. It occurs oftenest in males, in whom it is right-sided as a rule; in females usually it is left-sided.

The pathological changes which take place consist in a thickening of the coats of the sinus, desquamation of its endothelial lining, and formation of a thrombus, which, being infective, rapidly undergoes disintegration, and thus small emboli, teeming with micro-organisms, are carried away in the blood stream, and become impacted in such organs as the lungs, liver, and kidneys, setting up embolic abscesses. The prognosis without operative interference is very hopeless. The value of Horsley's suggestion of ligaturing the internal jugular vein is emphasised. Several cases are quoted at length.

[9] Berl. Klin. Woch, No. 15, 16, 1895. [10] Internat. Clinics, Vol. IV., No. 3. [11] Med. Chronicle, Oct. 1894. [12] Internat. Med. Mag., Ap., 1895. [13] Med. Week, Mar. 22. 1895. [14] Ann. Surg., May, 1895. [15] N.Y. Med. Record, Mar. 9, 1895. [16] Internat. Med. Mag., May, 1895. [17] Berl. Klin. Woch, 1895. [18] Lancet, May 18, 1895. [19] Clin. Journ., May 8, 1895. [20] Lancet, Ap. 20, 1895.

